# Expression dynamics and genome distribution of osmoprotectants in soybean: identifying important components to face abiotic stress

**DOI:** 10.1186/1471-2105-14-S1-S7

**Published:** 2013-01-14

**Authors:** Ederson A Kido, José RC Ferreira Neto, Roberta LO Silva, Luis C Belarmino, João P Bezerra Neto, Nina M Soares-Cavalcanti, Valesca Pandolfi, Manassés D Silva, Alexandre L Nepomuceno, Ana M Benko-Iseppon

**Affiliations:** 1 Departament of Genetics/Biological Sciences Center, Federal University of Pernambuco, Recife, Pernambuco, CEP 50.670-420, Brazil; 2 Embrapa Soybean, Brazilian Agricultural Research Corporation, Londrina, PR, CEP 86001-970, Brazil

## Abstract

**Background:**

Despite the importance of osmoprotectants, no previous *in silico *evaluation of high throughput data is available for higher plants. The present approach aimed at the identification and annotation of osmoprotectant-related sequences applied to short transcripts from a soybean HT-SuperSAGE (High Throughput Super Serial Analysis of Gene Expression; 26-bp tags) database, and also its comparison with other transcriptomic and genomic data available from different sources.

**Methods:**

A curated set of osmoprotectants related sequences was generated using text mining and selected seed sequences for identification of the respective transcripts and proteins in higher plants. To test the efficiency of the seed sequences, these were aligned against four HT-SuperSAGE contrasting libraries generated by our group using soybean tolerant and sensible plants against water deficit, considering only differentially expressed transcripts (p ≤ 0.05). Identified transcripts from soybean and their respective tags were aligned and anchored against the soybean virtual genome.

**Results:**

The workflow applied resulted in a set including 1,996 seed sequences that allowed the identification of 36 differentially expressed genes related to the biosynthesis of osmoprotectants [Proline (*P5CS*: 4, *P5CR*: 2), Trehalose (*TPS1*: 9, *TPPB*: 1), Glycine betaine (*BADH*: 4) and *Myo-*inositol (*MIPS*: 7, *INPS1*: 8)], also mapped *in silico *in the soybean genome (25 loci). Another approach considered matches using Arabidopsis full length sequences as seed sequences, and allowed the identification of 124 osmoprotectant-related sequences, matching ~10.500 tags anchored in the soybean virtual chromosomes. Osmoprotectant-related genes appeared clustered in all soybean chromosomes, with higher density in some subterminal regions and synteny among some chromosome pairs.

**Conclusions:**

Soybean presents all searched osmoprotectant categories with some important members differentially expressed among the comparisons considered (drought tolerant or sensible *vs*. control; tolerant *vs*. sensible), allowing the identification of interesting candidates for biotechnological inferences. The identified tags aligned to corresponding genes that matched 19 soybean chromosomes. Osmoprotectant-related genes are not regularly distributed in the soybean genome, but clustered in some regions near the chromosome terminals, with some redundant clusters in different chromosomes indicating their involvement in previous duplication and rearrangements events. The seed sequences, transcripts and map represent the first transversal evaluation for osmoprotectant-related genes and may be easily applied to other plants of interest.

## Background

Osmoprotectants figure among the most fundamental solutes in living organisms, being present from bacteria and fungi to higher plants and animals [1]. Main plant osmoprotectants are chemically composed by amino acids or carbohydrates, but share common features as low molecular weight and nontoxic character even at high concentrations, playing vital roles during abiotic stresses in plants as salinity, drought and chilling [2].

To face such constraints many plants accumulate organic osmolytes, or compatible solutes, in response to the resulting osmotic stress, maintaining cell turgor and therefore the driving gradient for water uptake. They include sugars, mainly fructose and sucrose, sugar alcohols (like *Myo-*inositol), complex sugars (like trehalose and fructans) and charged metabolites (like glycinebetaine, proline and ectoine) [1,3].

Osmolytes can also act as free-radical scavengers or chemical chaperones by directly stabilizing membranes and/or proteins [4]. Moreover, the accumulation of compatible solutes may also protect plants against damage by scavenging of reactive oxygen species, and by their chaperone-like activities in maintaining protein structures and functions [5]. Plant cells defend against stresses by modulating their expression according to the type and severity of stress and developmental stage of the plant [6].

Most previous works focused on expression assays regarding a single osmoprotectant as in Chen *et al. *[7] or searches in EST databases as in Barros *et al. *[8] or even their expression evaluation in transgenic plants [9,10]. No previous appreciation regarding in deep evaluation of transcriptomics databases generated under stress with Next Generation Sequencing (NGS) was carried out up to date. In the present work an 'in silico' annotation workflow was carried out integrating high throughput transcriptomics in soybean (*Glycine max*) plants under water deficit and biotic stress using HT-SuperSAGE, as compared with traditional transcriptomics and genome distribution of plant osmoprotectants.

The present approach focused on seven genes related to the biosynthesis of four classes of the most important plant osmoprotectants: Proline (genes *P5CS *and *P5CR*), Trehalose (*TPS1 *and *TPPB*), Glycine betaine (*BADH *and *CMO*) and *Myo-*inositol (*INPS1*).

**Proline **- Comprises a proteinogenic amino acid, essential for primary metabolism in plants during drought and salt stresses, presenting a molecular chaperone role due to its stabilizing action either as a buffer to maintain the pH of the cytosolic redox status of the cell [11] or as antioxidant through its involvement in the scavenging of free highly reactive radicals [12] or still acting as a singlet oxygen quencher [13]. In higher plants, proline biosynthesis may proceed either via glutamate, by successive reductions catalyzed by Delta(1)-pyrroline-5-carboxylate synthase (P5CS) and Delta(1)-pyrroline-5-carboxylate reductase (P5CR) or by ornithine pathway, by ornithine d-aminotransferase (OAT), representing generally the first activated osmoprotectant after stress perception [14,15].

**Trehalose **- In plants this sugar participates mainly in the response to dehydration being first described in the so called resurrection plants *Myrothamnus flabellifolius *[16] and *Selaginella tamariscina *[17] both able to recover after almost complete dehydration. Such ability to act in the stabilization of proteins and membranes [18], as well as its role in ROS scavenging process [19] are the possible features of its cellular function during non-ideal conditions encountered by plants, where it's synthesis normally occurs by the formation of the trehalose-6-phosphate (T6P) from the UDP-glucose and glucose-6-phosphate, a reaction catalyzed by the trehalose 6-phosphate synthase (TPS). Afterwards the T6P is dephosphorylated by the trehalose-6-phosphate phosphatase (TPP) resulting in the formation of free trehalose [20]. A transgenic assay using *Agrobacterium*-mediated gene transfer allowed the insertion of the gene *TPS1 *from yeast to tomato plants and resulted in higher content of chlorophyll and starch, besides pronounced tolerance to drought, salinity and oxidative stress, despite some pleiotropic changes [21].

**Glycine betaine (GB) **- Regards a quaternary ammonium compound (QAC) occurring in plants, animals and microorganisms. According to Chen and Murata [22] GB accumulates in chloroplasts and plastids especially in halotolerant plants, but also in other plants under high salinity, drought and cold stresses [23], with a recognized role associated to antioxidative responses [24]. In most organisms GB is synthesized either by the oxidation (or dehydrogenation) of choline or by the N-methylation of glycine. However, the pathway from choline to GB has been the main GB-accumulation pathway in plant species [25]. In this pathway choline is converted to betaine aldehyde by choline monooxygenase (CMO) [26], which is then converted to GB by betaine aldehyde dehydrogenase (BADH) [27].

***Myo*-inositol - **This osmoprotectant is an important cellular component forming the basis of a significant number of lipid signaling molecules involved in diverse pathways, including stress responses. *Myo-*inositol is the most abundant stereoisomer among the nine existing in nature, composed by a cyclohexanehexol, which is a cyclic carbohydrate with six hydroxyl groups, one on each carbon ring [28], acting as substrate in the biosynthesis of many compounds, especially the raffinose family oligosaccharides (RFOs) [29] that accumulate in plants under stress conditions [30]. In multicellular eukaryotes, *Myo-*inositol becomes incorporated into phosphatidylinositol phosphate (PtdInsP), *Myo-*inositol phosphate (InsP), and certain sphingolipid signalling molecules that act in diverse processes, including regulation of gene expression [31]. It is synthesized by a two-step pathway, including: (1) conversion of D-glucose-6-P to D-*Myo-*inositol (1)-Monophosphate, 1D-MI-1-P, which is catalyzed by a L-*Myo-*inositol 1-phosphate synthase (MIPS) [32], and (2) specific dephosphorylation to free *Myo-*inositol by the Mg^++ ^dependent L-*Myo-*inositol 1-phosphate phosphatase (IMP) [33]).

Considering the potential of these molecules for plant biotechnological approaches, the present work generated a curated list of osmoprotectants, osmoprotectant-related sequences and important regulatory elements, indicating most adequate tools for their identification and annotation. To evaluate the sensitivity of the proposed approach, the generated seed sequences and the proposed workflow were used to search of osmoprotectant-related sequences in short sequences (26 bp) generated from HT-SuperSAGE [34] deposited in the GENOSOJA (Brazilian Soybean Genome Consortium) data Bank [35]. A significant number of tags matched to known osmoprotectant-related sequences showing the effectiveness of the present approach useful for searches in other (actually very abundant) databanks comprising second generation sequences associated to the high performance sequencing approaches [e.g. Pyrosequencer (454 Roche^®^), Solexa (Illumina^®^) and SOLiD (Applied Biosystems^®^)] regarding genomic and transcriptomic libraries.

The present work also represents the first overall evaluation of the osmoprotectants in a higher plant comparing the prevalence of genes encoding enzymes of osmoprotectants biosynthetic pathways in sequence databanks with different backgrounds considering tissues, stages, stress conditions and also molecular approaches used to generate transcripts (ESTs, subtractive, cDNA full length, HT-SuperSAGE, BACs, etc.). In this aspect soybean offers one of the most abundant data sources for such an evaluation in legumes (see Benko-Iseppon *et al. *[36]), due to its importance as a source of food and oil in our planet.

## Results and discussion

### Seed sequences and annotation routine

**Figure 1:**
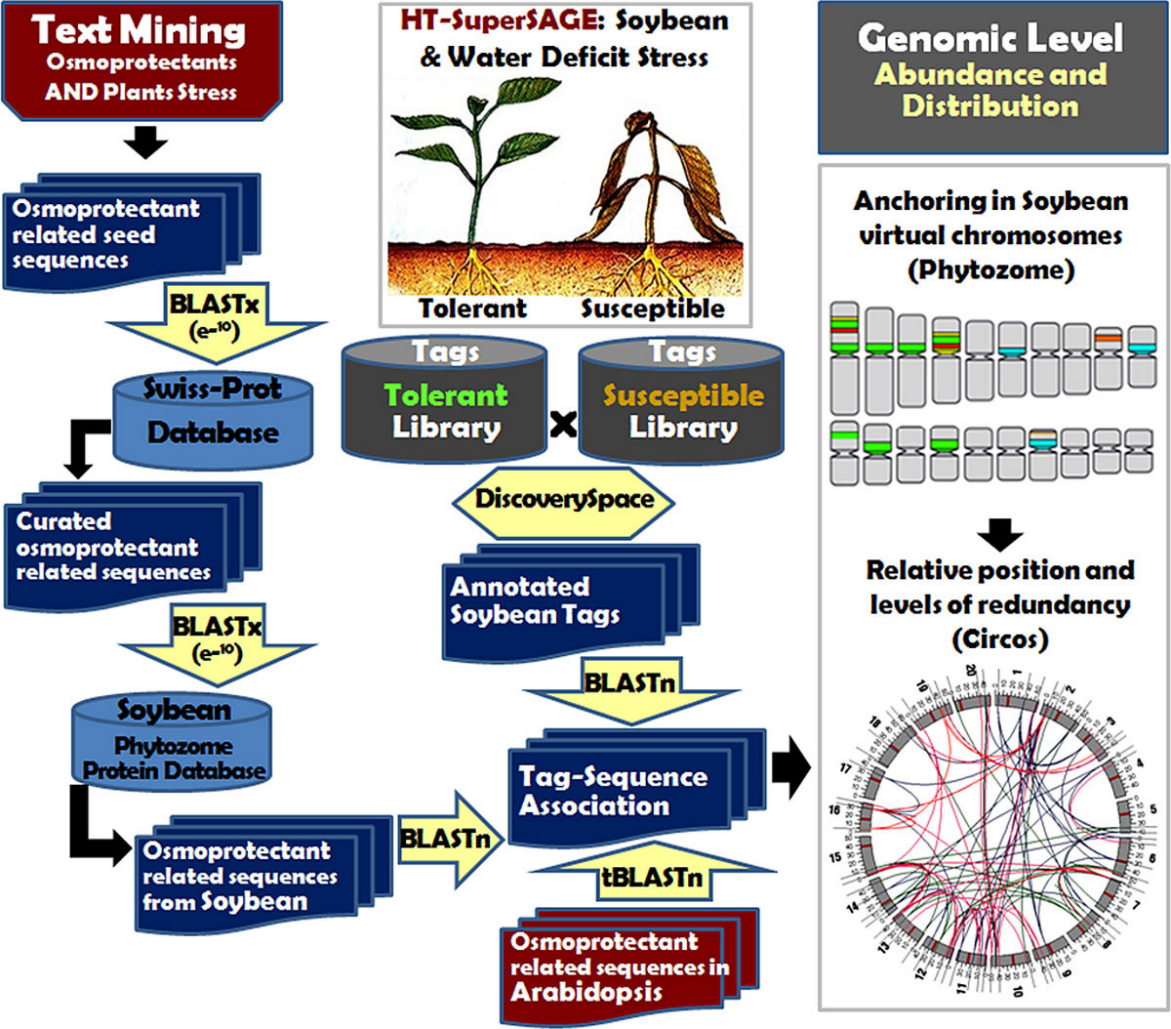
**Schematic representation of the *in silico *steps for the generation of the local bank of seed sequences, their annotation, SuperSAGE tag-gene association, expression evaluation and anchoring of osmoprotectant candidates and their matched tags in the soybean virtual chromosomes**.

The strategy regarding the use of seed sequences to find relevant literature and posterior mining and curation (Figure 1) was very effective, allowing the identification of 1,996 seed-sequences (Additional file 1) related to the procured osmoprotectants (proline, trehalose, *Myo-*inositol and glycine betaine). The sequences were aligned (BLASTx, cutoff e^-10^) against the soybean peptide database at Phytozome v8.0 [37], also permitting the identification of the respective transcripts from soybean transcriptome used to associate with the transcripts from HT-SuperSAGE libraries.

### Selection of HT-SuperSAGE tags for expression evaluation

After exclusion of the singlets, 2,551,286 tags from four libraries were selected for further evaluation, concerning 120,770 unitags. Considering the contrasts between any pair of libraries compared the number of unitags per library ranged from 73,807 to 89,205. The numbers of differentially expressed tags [upregulated (UR) and downregulated (DR) at the level of p ≤ 0.05] for each compared pair of libraries are presented in the Table 1. In all analyzed contrasts, the number of tags differentially expressed overruled the observed amount in the comparison among the controls. The same situation was observed in relation to the UR and DR tags, highlighting the effect of the stress application in the gene activation in both accessions, indicating a richness of analyzable transcripts in the present approach.

**Table 1:** Tags from soybean drought tolerant and sensible accessions, considering contrasting libraries and their expression profile.

Contrast	TS *vs*. TC	SS *vs*. SC	TS *vs*. SS	TC *vs*. SC	Total
UR	13,532	10,751	12,347	6,468	43,098
DR	7,423	5,587	7,634	3,135	23,779
n.s.	53,878	72,867	53,826	73,067	253,638
Unitags	74,833	89,205	73,807	82,670	320,515

UR: upregulated HT-SuperSAGE tags (p ≤ 0.05, DiscoverySpace 4.01); DR: downregulated tags; n.s.: not significant; TS: drought-tolerant Embrapa 48 roots under water deficit; TC: drought-tolerant Embrapa 48 root without stress; SS: drought-sensible BR-16 roots under water deficit; SC: drought-sensible BR-16 roots without stress).

### Identification of osmoprotectant-related genes and differential expression in soybean

The carried approach was very successful, allowing the identification of 36 differentially expressed HT-SuperSAGE tags associated to 65 osmoprotectant-related sequences anchored in 25 loci (Glyma sequences; Additional file 2) based on the generated seed sequence bank (Additional file 1). Many of them regard interesting candidates for a posterior in deep evaluation, as further discussed.

### Betaine aldehyde dehydrogenase (BADH, EC 1.2.1.8)

A total of 77 osmoprotectants OSMTL sequences presented significant similarity (BLASTx, e-value cut-off e^-10^) to *G. max *sequences from four loci coding BADHs and annotated as Aldehyde Dehydrogenase Family 10A (Additional file 1). Out of four loci, one (Glyma06g19820) was associated with four HT-SuperSAGE tags in BLASTn alignments, tolerating at most a single mismatch (Additional file 2). From these, two (GmDr_44 and 3640) were induced after stress in both tolerant and sensible accessions (Embrapa 48 and BR-16), being mapped in the 3'UTRs of all three alternative transcripts of the locus Glyma06g19820 (Additional file 2). Other two tags (GmDr_2643 and 55655) were induced only in the drought sensible accession BR-16 after stress regarding the same three transcripts, whereas one of them (GmDr_2443) was mapped in the 3'UTR and another in the CDS (GmDr_55655) (Additional file 2). The tag GmDr_55655 also mapped in the transcript Glyma11g27100.1 with a mismatch in the CDS region, but no 3'UTR was identified for this transcript (Additional file 2). Despite its induction in the sensible accession in relation to the control, the normalized frequency was only six tmp (tags per million; Additional file 2). Thus, the locus Glyma06g19820 emerged as a likely BADH candidate gene induced in response to the water deficit stress in the studied libraries.

The members of the ALDH (aldehyde dehydrogenase) gene superfamily here identified in soybean genome were also categorized by Kotchoni *et al. *[38] that provided a unified nomenclature for the soybean ALDH members, including the ALDH family 10, also described as putative BADH. A previous work [39] also observed the induction of BADH (almost 8-fold and 2-fold increase) under salinity and its accumulation in response to water stress or drought, indicating a common response of the plant to osmotic changes that affect its water status. The importance of identifying different candidates of this enzyme was highlighted by Nakamura *et al. *[40] that isolated two BADH transcripts (BBD1 and BBD2) from barley, one of them (BBD2) more similar to previously reported BADH genes from dicots. Both barley BADH genes showed different expression patterns. While BBD1 transcript was more abundant in roots and was induced to higher levels under salinity, drought and abscisic acid (ABA) treatment, the BBD2 transcript was more abundant in leaves after induction by salt, drought, PEG and ABA treatments, showing the potential of both genes for breeding purposes.

### Delta(1)-pyrroline-5-carboxylate synthase (P5CS, EC 1.5.1.12)

Polypeptides regarding seven transcripts of delta(1)-pyrroline-5-carboxylate synthase 2 were similar to 19 OSMTL sequences (Additional file 1). Considering the transcripts, only Glyma18g40770.1 was not linked to a SuperSAGE tag. Seven tags matched with transcripts of the remaining six loci. From these, four were differentially expressed in the stressed library as compared with the negative control: one DR (downregulated GmDr_18680 mapped in the 3'UTR of Glyma01g24530.1) in both tolerant and sensible accessions; two DR tags in the sensible accession (tag GmDr_4918 mapped in the last CATG of the CDS of Glyma02g41850.1 and in the CDS of Glyma14g07120.1, and also tag GmDr_20800 at the 3'UTR of Glyma07g16510.1), besides a UR tag (FC = 9.6) only in the tolerant accession (tag GmDr_57499 at the CDSs of Glyma02g41850.1 and Glyma14g07120.1) (Additional file 2). The fact that both tags were associated to the CDS of Glyma02g41850.1 (Additional file 2) may be justified by the absence of the CATG sequence in the 3'UTR region. Even in the absence of expressive induction, the most prevalent tag (19-40 tpm; GmDr_4918) was observed in all libraries (Additional file 2).

Significant upregulation (RTqPCR) in leaves of PvP5CS (from common bean *Phaseolus vulgaris*) was demonstrated with transcription increase after 4d drought stress (2.5 times the control level), 2 h post-treatment (200 mM NaCl) of salt stress (about 16.3 times the control) and 2 h after of cold stress (11.7-fold). Another P5CS (PvP5CS2) also from common bean [41] presented predicted amino acid sequence showing 83.7% identity with PvP5CS and an overall 93.2% identity with GmP5CS [*G. max *P5CS], suggesting PvP5CS2 represented a soybean P5CS homolog gene. Indels (insertion and deletion events) and SNPs (single nucleotide polymorphisms) were found in the cloned PvP5CS2 genome sequence when the authors compared different accessions, helping in the development of a molecular marker in the chromosome b01. The association of molecular markers and phenotypes, in this case Pro accumulation is highly applicable for genetic improvement of plants and germplasm screening.

### Delta(1)-pyrroline-5-carboxylate reductase (P5CR, EC 1.5.1.2)

The seed sequences OSMTL431, 432 and 434 were similar to P5CR polypeptides from two soybean loci (Glyma19g31230 and Glyma03g28480; Additional file 1), which transcripts were associated to SuperSAGE tags (Additional file 2). Of these one was repressed in the sensible accession (GmDr_4445, mapped at the 3'UTR region of the transcript Glyma19g31230.1) after stress (Additional file 2). A second one (GmDr_42728, mapped in the 3'UTR of Glyma03g28480.1 and CDS of Glyma03g28480.1) was not significantly modulated in the tolerant accession under stress as compared with the respective control, but presented a significant difference when compared to the sensible accession under stress (fold change of 12,0) (Additional file 2).

Previous genomic analysis indicated that there are only two to three copies of the P5CR gene in the soybean genome [42], similar to the proposed for pea [43]. Besides, the primary structure of pea P5CR is 85% identical with that of soybean isolated by Delauney and Verma [42]. The mentioned pea P5CR exhibited significant homology to human, yeast, and *E. coli *P5CR [43], a conservation that favours the here used approach in the search of orthologs using seeds sequences.

The suggestion that P5CR gene is osmoregulated was confirmed after subjecting soybean seedlings to osmotic stress (400 mM NaCl solution), resulting in an almost six-fold increase in the level of root P5CR mRNA [42]. An interesting aspect in association with proline overexpression and accumulation regards its influence on the concentration of other amino acids, suggesting a coordinated regulation of distinct metabolic pathways [44]. Free amino acid levels were compared in wild type and transgenic soybean (*G. max *cv. Ibis) transformed with P5CR in sense and antisense directions. The most rapid increase in Pro content was found in the sense transformants that exhibited the least water loss, while the slowest elevation of Pro levels was detected in the antisense transformants that exhibited the greatest water loss during stress. Correspondingly, the level of the Pro precursors Glu and Arg was higher in sense transformants and lower in antisense ones compared to the wild type plants during the initial exposure to stress (drought and heat) [44].

### *Myo-*inositol 1-phosphate synthase (MIPS, EC 5.5.1.4)

A total of 13 OSMTL seed sequences (Additional file 1) presented similarities to polypeptides from three soybean *Myo-*inositol sequences. With exception of the transcript Glyma08g14670.1 that matched with MIPS1 the other two transcripts, matching MIPS2 and MIPS3, were associated to tags (Additional file 2). The tag GmDr_37 (mapped at the 3'UTR in all four alternative transcripts of Glyma18g02210) was the most frequent tag (615-1446 tpm) being DR in both stressed accessions (Additional file 2). The other tag (GmDr_3907) presented a perfect match with the 3'UTR of all three alternative transcripts of Glyma05g31450, with DR expression in the sensible accession under stress (Additional file 2). Another tag, GmDr_5821 (mapped at the 3'UTR in four alternative transcripts of Glyma18g02210) was induced (UR) in the tolerant accession Embrapa 48 under stress when compared with the respective control (Additional file 2). Considering all transcripts identified the locus Glyma18g02210 (MIPS2) seems to be the most interesting candidate for future validation and transgenic expression (in detriment to Glyma05g31450, MIPS3).

The confirmation of such a differential expression regarding MIPS is useful for plant breeding as highlighted by Kaur *et al. *[45] that observed two divergent genes encoding MIPS1 and MIPS2 (isolated from a drought-tolerant plant) in chickpea with differential expression but discrete overlapping roles, despite their pronounced divergence in respect to their íntrons composition, at the same time retaining 85% identity to their exons. Expression analysis showed both genes being expressed in all organs except seed, where only MIPS2 transcript was detected. Under environmental stresses (high temperature and salinity), only MIPS2 was induced whereas MIPS1 expression remained the same. Also, in those conditions of high temperature and salinity MIPS2 retained higher activity than MIPS1.

### *Myo-*inositol monophosphatase (IMP, EC 3.1.3.25)

A total of 12 seed sequences (OSMTL61-66, OSMTL331-335 and OSMTL94) presented similarities with annotated IMP polypeptides regarding 10 *G. max *loci (Additional file 1), for those 19 SuperSAGE tags were identified. From the differentially expressed tags (Additional file 2), GmDr_3452 mapped at the last CATG of the Glyma08g19430.1, with bases in the CDS and 3'UTR and was induced after stress in both accessions. Similarly other tags were induced in the tolerant accession under stress (GmDr_23844, at the 3'UTR of Glyma16g28310.1 and GmDr_32375 at the 3'UTRs of both Glyma07g30110.1 and Glyma08g07200.1) (Additional file 2). By the other hand, the tag GmDr_5543 was mapped at the 3'UTR of three alternative transcripts of Glyma04g01170, being upregulated in the sensible accession and downregulated in the tolerant accession under stress (Additional file 2). Also the tag GmDr_25343 (mapped at the 3'UTR of Glyma15g07240.1) was downregulated in the tolerant accession after stress (Additional file 2). The abundance and differential expression of various IMP candidates in diverse comparisons indicate an important role in soybean water deficit. Despite of that and of the known role of these osmoprotectant-related genes, it is interesting that few expression essays or transgenic approaches have been carried using these candidates up to date.

In Arabidopsis transformants [46], two IMP candidate genes, IMPL1 and IMPL2 were expressed in a similar manner both in the vegetative and reproductive organs. The expression of IMP genes in a promoter-GUS assay on developing seeds was not coupled with the expression of the genes encoding MIPSs, which supply the substrate for IMPs in a 'de novo' synthesis pathway. Instead, IMP expression was correlated with SAL1 expression (encoding *Myo-*inositol polyphosphate 1-phosphatase), which is involved in the *Myo-*inositol salvage pathway.

### Trehalose-6-phosphate synthase (TPS, EC:2.4.1.15)

After BLASTx 53 TPS OSMTL sequences were associated with 26 soybean transcripts of 21 loci (Additional file 1). From these, tags matched 22 transcripts and 17 loci, including TPS5, TPS7, TPS9 and TPS11 (Additional file 1). Among the differentially expressed tags (Table S2), three (GmDr_1203, GmDr_3893 and GmDr_9994) mapped at Glyma01g03870.1, Glyma06g19590.1 and Glyma17g07530, respectively and were considered induced in both accessions under stress. In turn, tag GmDr_62319 (Glyma04g35190.1, 3'UTR) was induced only in the sensible accession, while tag GmDr_25843 (Glyma01g03870.1, 3'UTR) was repressed under stress in the tolerant accession (Additional file 2).

Other two tags (GmDr_48598 and GmDr_57367, both mapping in Glyma01g03870.1, 3'UTR) were also DR in the tolerant accession under stress (Additional file 2). These two tags with different expression behavior for the same transcript could be considered as a possible annotation mistake, but further analysis showed that they regard sister tags, differing by a SNP, both mapping to Glyma01g03870.1 in an upstream site when compared to the mapped GmDr_25843 tag. Therefore, this last tag could be the result of a partial *Nla*III digestion, with the DR expression being questionable and therefore demanding validation. By the other hand, this possibility is quite unlikely, since a double digestion with *Nla*III was carried out prior to generation of HT-SuperSAGE libraries.

A similar situation was observed for two tags (GmDr_169137 and GmDr_198028, mapped both at Glyma06g19590.1) considered UR in the tolerant accession, while other two UR tags (GmDr_53228 and GmDr_61653) aligned to the same transcript with a single mismatch (Additional file 2). A careful analysis revealed that the tags GmDr_169137, GmDr_198028 and GmDr_53228 mapped to CDS region, while GmDr_61653 mapped at the 3'UTR, in a CATG near the Poli-A tail, as expected for most SuperSAGE tags (Additional file 2). Thus, the most valid representative of this transcript seems to be GmDr_61653, induced in the tolerant accession under stress (Additional file 2).

Additional differentially expressed tags included GmDr_80395 (Glyma10g41680, 3'UTR) considered UR in the tolerant accession under stress; GmDr_66719 (mapped with two alternative transcripts of Glyma17g07530 at 3'UTR) UR in the sensible accession; GmDr_9508 (Glyma06g42820 and Glyma12g15500, both at CDS region), DR in the tolerant accession under stress (Additional file 2).

Such abundance and induction of TPS were also observed in other species. For example rice (*Oryza sativa*) contains 11 OsTPS genes, but only OsTPS1 showed TPS activity [47]. To demonstrate the physiological function of OsTPS1 the authors used the respective gene to transform rice plants and found that OsTPS1 overexpression improved the tolerance of seedling to cold, high salinity and drought conditions without other significant phenotypic changes.

### Trehalose-phosphatase family protein (TPP; EC 3.1.3.12)

Contrasting with the results generated for TPS, a single transcript was observed for TPP (Glyma04g41640.1) in a locus associated to sixteen available OSMTL sequences (Additional file 1). This transcript was associated to only two differentially expressed tags (GmDr_43033 and GmDr_108104), both mapped at the 3'UTR region (Additional file 2), with discrete expression (2-5 tpm) in two out of four libraries. As in our case, few examples in the literature associated TPP expression with water deficit stress in plants, maybe due to their restricted prevalence in previously analyzed libraries. Despite the scarce number of reports the work of Ge *et al. *[48] revealed the transient upregulation of OsTPP1 (rice) after salt, osmotic and abscisic acid (ABA) treatments, with discrete upregulation under cold stress. Also, the overexpression lines analysis revealed that OsTPP1 triggered abiotic stress response genes, suggesting a possible transcriptional regulation pathway in stress induced reprogramming initiated by OsTPP1.

### Tag-gene anchoring in the soybean genome

**Figure 2:**
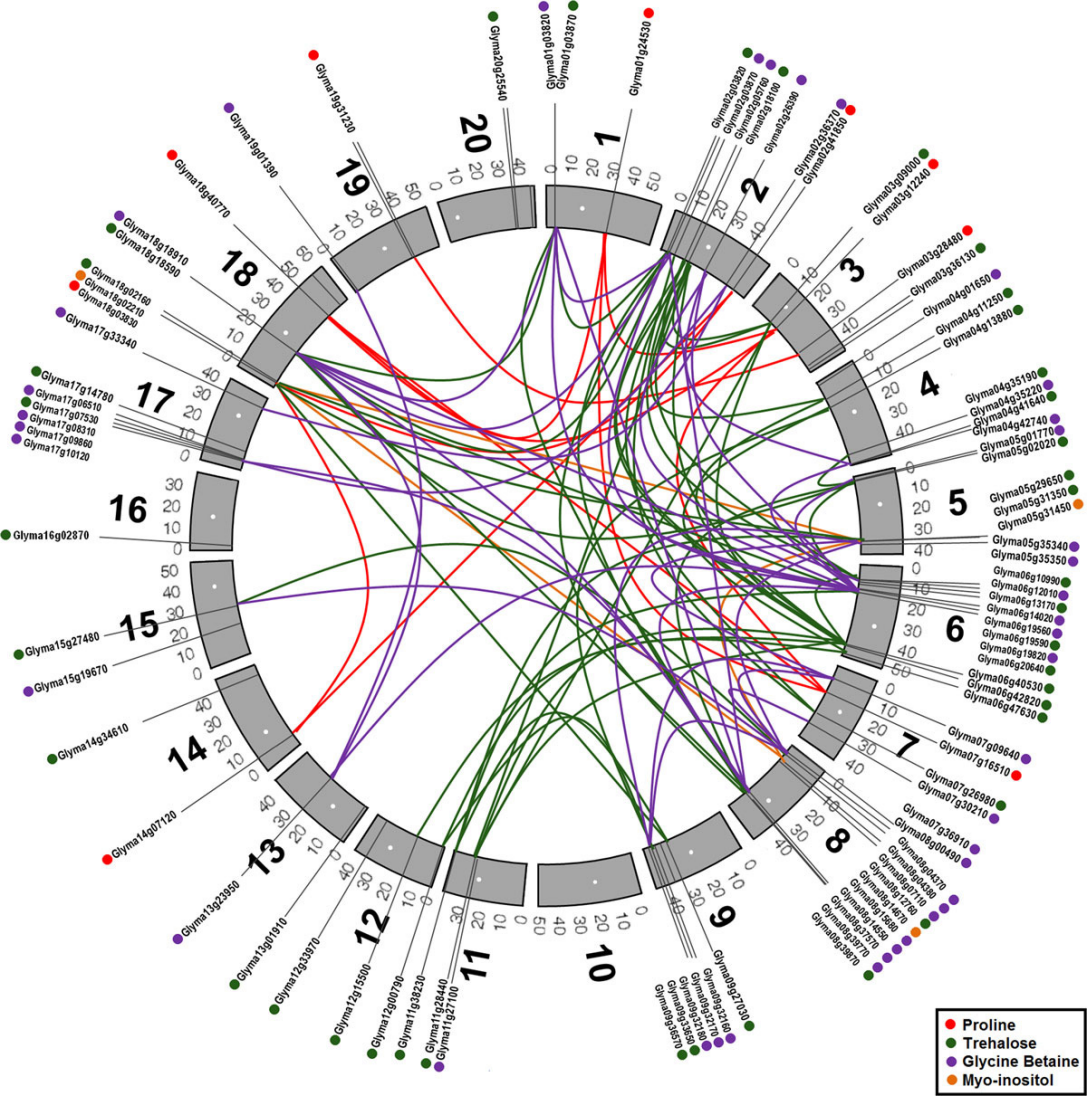
**Distribution and syntenic relationships of 124 osmoprotectants in soybean genome**. Chromosomes are marked at centromere (white circle) in their expected position. Each osmoprotectants name is shown outside in described position of SoyBase. Syntenic relationships are shown as color lines between chromosome regions. Red line - Proline (*P5CS *and *P5CR*); Green line - Trehalose (*TPS1 *and *TPPB*); Purple line - Glycine Betaine (*BADH *and *CMO*); Blue line - Cysteine (*OASTL *and *SAT*); Orange line - *Myo-*inositol (*INPS1*).

The search for osmoprotectant-associated homologs in soybean genes that matched with SuperSAGE tags recovered 179 sequences. However, these sequences were anchored in only 124 loci in the soybean genome (Additional file 3), what indicates the occurrence of alternative splicing of primary transcripts, suggesting an important role of osmoprotectants in vital processes during abiotic stresses in plants, probably inducing specific transcripts for particular environmental conditions. The anchoring of these osmoprotectant-related sequences and their respective SuperSAGE tags in the soybean virtual chromosomes revealed that osmoprotectant-related genes are present in 19 out of 20 soybean chromosomes (Figure 2). Most osmoprotectant-related sequences presented syntenic regions among non homologous chromosomes, often forming gene clusters mainly on both arms of chromosomes 2, 6 and 8, followed by chromosomes 5 and 17. On the opposite, some chromosomes presented few copies, as in the case of chromosomes 11-16 and 19-20, whereas chromosome 10 presented no match. The great number of osmoprotectant-related members in the short arm of chromosome 2 is also accompanied by a preferential distribution in the subterminal region, a phenomenon also observed in a lesser extent in the chromosomes 1, 5, 6, 7, 8, 9 and 17. Less frequently, gene-rich regions were also in the pericentromeric regions (e.g. in chromosomes 2, 3, 11 and 18) or intercalary regions.

A similar distribution was observed in regard to aquaporin genes, another gene family associated to drought stress in soybean [49]. Besides redundancies among chromosomes, aquaporins were also prevalent in terminal and subterminal gene clusters. As for aquaporins, the observed redundancy of osmoprotectant-related gene clusters corroborates previous suggestions of the soybean octoploid nature [50].

Another previous approach anchoring 59 soybean defense genes (two super-families: *R *resistance and *PR *pathogen related genes) in the virtual chromosomes of the legume *Medicago truncatula *revealed 1,253 sites, most of them clustered in subterminal or terminal positions. The 59 sequences were distributed in all nine medicago chromosomes, whereas 58 genes presented similarities with distinct segments in the same chromosome or appeared twice in distinct chromosomes [51]. Similar clustering was described for arabidopsis [52], indicating that such a distribution may occur in regard to different gene families and plant groups.

The redundancies observed probably reflect past duplication events, increasing the number of osmoprotectant-related genes in soybean genome [53,54]. The observed clustering and prevalence in some chromosomes, especially those combining different gene categories (as in the short arm of chromosomes 2, 6, and 7 or in the long arms of chromosomes 6, 8 and 9) indicate that these regions probably regard QTLs (Quantitative Trait Loci) useful for mapping approaches and marker assisted selection.

## Conclusions

High throughput sequencing is generating a huge amount of sequences in given tissues and under contrasting conditions. In the present case we evaluated osmoprotectant-related sequences in 26-bp tags from HT-SuperSAGE libraries from soybean coupled with Solexa/Illumina^® ^sequencing in a digital gene expression profile. The approach permitted tags identification and annotation and their association with sequences from different sources (genomic regions, transcripts and proteins); identifying 36 differentially expressed osmoprotectant-related transcripts relative to 25 loci potentially active comprising four osmoprotectants classes. The 1,996 seed sequences and the workflow are also applicable to evaluate other angiosperms. Their clustering observed in soybean may be prevalent in other plant groups (or at least in legumes) and may be associated to interesting QTLs for breeding purposes or still for metabolic engineering in association with drought and salinity and chilling tolerance.

## Methods

### Seed sequences and annotation routine

The selection of seed sequences (Additional file 1) was based in a literature search in the PubMed database [55] using the key words "Osmoprotectants" AND "Plant Stress". In the selected articles the NCBI [56] descriptors for posterior mining were selected and retrieved from the Uniprot SwissProt (cutoff e^-10^) using BLASTx. In order to confirm their involvement in the biosynthesis of osmoprotectants (proline, trehalose, *Myo-*inositol and glycine betaine) the sequences were aligned (BLASTx, cutoff e^-10^) against the soybean peptide database at Phytozome v. 8.0 [37], also allowing the identification of the respective transcripts from soybean transcriptome used to associate with the available SuperSAGE tags.

**Biological material, experimental design and stress application - **Soybean HT-SuperSAGE libraries were generated according to the procedures described by Matsumura *et al. *[57] at GenXPro GmbH, with posterior SOLEXA sequencing of the tags. The generated tags are distributed into four libraries (Additional file 2) including root tissues subjected to dehydration: two libraries from the drought tolerant cultivar Embrapa 48 [Tolerant after stress (TS) and negative control (TC)] and two libraries from a drought sensible cultivar BR-16 [Sensible after Stress (SS) and negative control (SC)]. The conditions for the generation of the mentioned libraries, time frame experiments, and laboratory protocols used are described in Soares-Cavalcanti *et al. *[58]. The generated sequences are available at the GENOSOJA database (Brazilian Soybean Genome Consortium) [35].

**Statistical analysis, tag-gene annotation and the tag fold change estimation - **The in silico procedures are illustrated in Figure 1. Initially 26 bp-tags were analyzed with the DiscoverySpace (v.4.01) software [59] aiming to identify unique tags (unitags) and those unitags differentially expressed (p ≤ 0.05) considering a contrast among two libraries. Tags counted only once (singlets) were excluded from the present evaluation. Unitags were annotated by BLASTn [60] against nucleotide sequences from the soybean Phytozome database v8.0 (Glyma1 cDNA dataset) [37,50]. BLASTn alignments (tag-hit) with e-values of 0.0001 or less and tolerating a single mismatch maximum (TSM) were taken into account. Moreover, only plus/plus alignments without mismatches regarding the four first bases CATG were accepted, in order to guarantee the integrity of the SuperSAGE tag. Specific keyword searches on the original glyma annotations were performed looking for the transcripts and tags candidates. Values reflecting expression data (p-value and up- or down-regulation regarding each tag) were associated to the data matrix including the respective tag annotation, the normalized frequencies in the libraries and the fold change values (FC). FC estimative were based on the ratio (R) of the normalized frequencies of the tag in the contrast of the two libraries, where the 'zero' frequency was replaced by 'one'. When R > 1 the FC were directly considered and when R < 1 the FC = - 1/R. Negative FC values indicated repressed tags.

### Tag-gene identification and anchoring in the soybean genome

A further approach consisted in the identification and generation of a curated list consisting of seven genes related to the biosynthesis of four classes of osmoprotectants [i.e. Proline (genes *P5CS *and *P5CR*), Trehalose (*TPS1 *and *TPPB*), Glycine betaine (*BADH *and *CMO*) and *Myo-*inositol (*INPS1*)]. For this purpose a initial list was generated based on well known data from *Arabidopsis thaliana *(Additional file 3) used to identify corresponding sequences at SoyBase available on Phytozome [37,50], allowing the construction of a local database comprising complete soybean osmoprotectants for the alignment with the previously identified SuperSAGE tags and posterior anchoring in the SoyBase web server (consisting of pseudochromosomes from genome sequences including mainly BACs and molecular markers).

### Circos mapping

Sequence matches for the nine selected osmoprotectant-related genes were aligned against the SoyBase pseudochromosomes aiming to infer about their distribution in the virtual chromosomes available at SoyBase. BLAST algorithm parameters (score, e-value and percentage of identity) were adjusted to allow the anchoring of soybean sequences position along the soybean virtual chromosomes. Afterwards the identified anchoring positions were submitted to the Circos program [61] and so edited to generate a picture of higher resolution. This approach allowed the generation of a graph based on a circular organization of the soybean chromosomes (n = 20), allowing the identification of a virtual ideogram with linear distribution of the osmoprotectants identified, the associated SuperSAGE tags, as well as redundant portions.

## List of abbreviations used

ABA: abscisic acid; ALDH: aldehyde dehydrogenase; Arg: arginine; BACs: bacterial artificial chromosome; BADH: betaine aldehyde dehydrogenase; CDS: coding sequence; CMO: choline monooxygenase; DR: down-regulated; FC: fold change value; GB: glycine betaine; GENOSOJA: Brazilian Soybean Genome Consortium; Glu: Glutamic acid; Glucose-6-P: glucose 6-phosphate; HP-SA: high performance sequencing approaches; HSP70: 70 kilodalton heat shock proteins; HT-SuperSAGE: High Throughput Super Serial Analysis of Gene Expression; IMP: L-*Myo-*inositol 1-phosphate phosphatise; MIPS: L-*Myo-*inositol 1-phosphate synthase; NGS: next generation sequencing; OAS: O-acetyl-L-serine; OAS-TL: O-acetyl-L-serine thiol lyase; OAT*: *ornithine d-aminotransferase; OsTPS: *Oryza sativa *Trehalose-6-phosphate synthase; P5CR: delta(1)-pyrroline-5-carboxylate reductase; P5CS: delta(1)-pyrroline-5-carboxylate synthase; PEG: polyethylene glycol; Pro: Proline; PtdInsP: phosphatidylinositol phosphate; QTLs: quantitative trait loci; RT-qPCR: real-time quantitative PCR; SAL1: *myo- *inositol polyphosphate 1-phosphatase; SAT: serine acetyltransferase; SNP: single nucleotide polymorphism; T6P: trehalose-6-phosphate; TPP: trehalose-6-phosphate phosphatise; *TPPB: *Trehalose 6- phosphate phosphatase B; TPS: Trehalose-6-phosphate synthase; TSM: tolerating a single mismatch; UDP: Glycosyltransferase/trehalose-phosphatase family protein; UR: up-regulated; UTRs: untranslated region.

## Competing interests

The authors declare that they have no competing interests.

## Authors' contributions

EAK, VP and ALN generated the HT-SuperSAGE libraries. EAK and JRCFN carried out the identification of tags and differential expression analysis. RLOS, LCB, JPBN, NMSC and MDS generated and curated the seed sequence bank, while JPBN and AMBI generated the data for genome anchoring. AMBI coordinated the research.

## Declarations

The publication costs for this article were funded by the first author's institution.

This article has been published as part of *BMC Bioinformatics *Volume 14 Supplement 1, 2013: Computational Intelligence in Bioinformatics and Biostatistics: new trends from the CIBB conference series. The full contents of the supplement are available online at http://www.biomedcentral.com/bmcbioinformatics/supplements/14/S1.

## Supplementary Material

Additional file 1 **Table S1**. BLASTx (Identity % and e-value) results regarding nucleotide sequences involved in the biosynthesis pathway and corresponding best hit (cut-off e^-10^) in the Uniprot-SwissProt and Phytozome (v.8 *Glycine max*) databases, represented by the respective loci/transcripts in soybean (Glyma) as well as their annotation. Click here for file

Additional file 2 **Table S2**. BLASTn results (Identity %, e-value) using HT-SuperSAGE 26-bp tags against *Glycine max *seed sequences given in Table S1. Libraries consisted of soybean cultivar Embrapa 48, tolerant (T) against drought, with roots submitted to water deficit after 1-6 h of stress submission (TS) as compared with the tolerant non stressed control (TC). The same treatment was given to the drought sensible cultivar (BR-16) considering stressed (SS) and its respective control (SC). Tag annotation occurred against transcripts on the soybean Phytozome database. For each library normalized frequencies were considered (tpm: tags per million). Regarding comparisons among different treatments, fold change (FC), regulation (reg) [including upregulated (UR, in red) downregulated (DR, in green) and also non significant differential expression (ns), at 5% level] are given, as well as the tag-mapping position in the respective soybean transcript (Glyma). Click here for file

Additional file 3 **Table S3**. Main soybean transcripts similar to known osmoprotectants-genes (I.) from *Arabidopsis thaliana *(used as seed sequences). II. tBLASTn results and sequence evaluation of soybean Osmoprotectants-genes. Information available include number of hits, best match of each gene class and features of hits: size range (maximal and minimal) in nucleotides (n), ORF (Open Reading Frame) size range in amino-acids (aa), e-value range based on SoyBase web resource, as well as number of matching HT-SuperSAGE tags. Click here for file

## References

[B1] Yancey PH Organic osmolytes as compatible, metabolic and counteracting cytoprotectants in high osmolarity and other stresses J Exp Biol 2005 208 2819 2830 10.1242/jeb.01730 16043587

[B2] Rontein D Basset G Hanson AD Metabolic engineering of osmoprotectant accumulation in plants Metab Eng 2002 4 49 56 10.1006/mben.2001.0208 11800574

[B3] Serraj R Sinclair TR Osmolyte accumulation: can it really help increase crop yield under drought conditions? Plant Cell Environ 2002 25 333 341 10.1046/j.1365-3040.2002.00754.x 11841674

[B4] McNeil SD Nuccio ML Hanson AD Betaines and related osmoprotectants: targets for metabolic engineering of stress resistance Plant Physiol 1999 120 945 949 10.1104/pp.120.4.945 10444077 PMC1539222

[B5] Wang W Vinocur B Altman A Plant responses to drought, salinity and extreme temperatures: towards genetic engineering for stress tolerance Planta 2003 218 1 14 10.1007/s00425-003-1105-5 14513379

[B6] Nouri M-Z Toorchi M Komatsu S Sudaric A Proteomics Approach for identifying abiotic stress responsive proteins in soybean Soybean - Molecular Aspects of Breeding 2011 Croatia: Intech 187 214

[B7] Chen S Gollop N Heuer B Proteomic analysis of salt-stressed tomato (*Solanum lycopersicum*) seedlings: effect of genotype and exogenous application of glycinebetaine J Exp Bot 2009 60 2005 2019 10.1093/jxb/erp075 19336390 PMC2682497

[B8] Barros OS Soares-Cavalcanti NM Vieira-Mello GS Wanderley-Nogueira AC Calsa-Junior T Benko-Iseppon AM *In silico* evaluation of osmoprotectants in eucalyptus transcriptome Lect Notes Comput Sci 2009 5488 66 77 10.1007/978-3-642-02504-4_6

[B9] Holmström KO Somersalo S Mandal A Palva TE Welin B Improved tolerance to salinity and low temperature in transgenic tobacco producing glycine betaine J Exp Bot 2000 51 343 177 185 10.1093/jexbot/51.343.177 10938824

[B10] Kathuria H Giri J Nataraja KN Murata N Udayakumar M Tyagi AK Glycinebetaine-induced water-stress tolerance in codA-expressing transgenic indica rice is associated with up-regulation of several stress responsive genes Plant Biotechnol J 2009 7 6 512 526 10.1111/j.1467-7652.2009.00420.x 19490479

[B11] Verbruggen N Hermans C Proline accumulation in plants: a review Amino Acids 2008 35 4 753 759 10.1007/s00726-008-0061-6 18379856

[B12] Smirnoff N Cumbes QJ Hydroxyl radical scavenging activity of compatible solutes Phytochemistry 1989 28 1057 1060 10.1016/0031-9422(89)80182-7

[B13] Bhalu B Mohanty P Molecular mechanisms of quenching of reactive oxygen species by proline under stress in plants Curr Sci 2002 82 5 525 532

[B14] Savouré A Jaoua S Hua XJ Ardiles W Van Montagu M Verbruggen N Isolation, characterization, and chromosomal location of a gene encoding the delta 1-pyrroline-5-carboxylate synthetase in *Arabidopsis thaliana* FEBS Lett 1995 372 1 13 19 10.1016/0014-5793(95)00935-3 7556633

[B15] Parida AK Dagaonkar VS Phalak MS Aurangabadkar LP Differential responses of the enzymes involved in proline biosynthesis and degradation in drought tolerant and sensitive cotton genotypes during drought stress and recovery Acta Physiol Plant 2008 30 5 619 627 10.1007/s11738-008-0157-3

[B16] Drennan PM The occurence of trehalose in the leaves of the desiccation-tolerant angiosperm *Myrothamnus flabellifolius *Welw J Plant Physiol 1993 142 4 493 496 10.1016/S0176-1617(11)81257-5

[B17] Liu M-S Chien C-T Lin T-P Constitutive components and induced gene expression are involved in the desiccation tolerance of *Selaginella tamariscina* Plant Cell Physiol 2008 49 4 653 663 10.1093/pcp/pcn040 18326542

[B18] Fernandez O Béthencourt L Quero A Sangwan RS Clément C Trehalose and plant stress responses: friend or foe? Trends Plant Sci 2010 15 7 409 417 10.1016/j.tplants.2010.04.004 20494608

[B19] Luo Y Lib W-M Wang W Trehalose: protector of antioxidant enzymes or reactive oxygen species scavenger under heat stress? Environ Exp Bot 2008 63 1-3 378 384 10.1016/j.envexpbot.2007.11.016

[B20] Wingler A The function of trehalose biosynthesis in plants Phytochemistry 2002 60 5 437 440 10.1016/S0031-9422(02)00137-1 12052507

[B21] Cortina C Culiáñez-Macià FA Tomato abiotic stress enhanced tolerance by trehalose biosynthesis Plant Sci 2005 169 1 75 82 10.1016/j.plantsci.2005.02.026

[B22] Chen THH Murata N Glycinebetaine: an effective protectant against abiotic stress in plants Trends Plant Sci 2008 13 9 499 505 10.1016/j.tplants.2008.06.007 18703379

[B23] Jagendorf AT Takabe T Inducers of glycinebetaine synthesis in barley Plant Physiol 2001 127 1827 1835 10.1104/pp.010392 11743126 PMC133586

[B24] Chen THH Murata N Glycinebetaine protects plants against abiotic stress: mechanisms and biotechnological applications Plant Cell Environ 2011 34 1 1 20 10.1111/j.1365-3040.2010.02232.x 20946588

[B25] Weretilnyk EA Bednarek S McCue KF Rhodes D Hanson AD Comparative biochemical and immunological studies of the glycine betaine synthesis pathway in diverse families of dicotyledons Planta 1989 178 3 342 352 10.1007/BF00391862 24212901

[B26] Rathinasabapathi B Burnet M Russell BL Gage DA Liao PC Nye GJ Golbeck JH Hanson AD Choline monooxygenase, an unusual iron-sulfur enzyme catalyzing the first step of glycine betaine synthesis in plants: prosthetic group characterization and cDNA cloning Proc Natl Acad Sci USA 1997 94 7 3454 3458 10.1073/pnas.94.7.3454 9096415 PMC20391

[B27] Vojtechova M Hanson AD Munoz-Clares RA Betaine-aldehyde dehydrogenase from amaranth leaves efficiently catalyzes the NAD-dependent oxidation of dimethylsulfoniopropionaldehyde to dimethyl-sulfoniopropionate Arch Biochem Biophys 1997 337 1 81 88 10.1006/abbi.1996.9731 8990271

[B28] Dastidar K Maitra S Goswami L Roy D Das KP Majumder AL An insight into the molecular basis of salt tolerance of l-*myo-*inositol 1-P synthase (PcINO1) from *Porteresia coarctata *(Roxb.) *Tateoka*, a halophytic wild rice Plant Physiol 2006 140 1279 1296 10.1104/pp.105.075150 16500989 PMC1435794

[B29] Karner U Peterbauer T Raboy V Jones DA Hedley CL Richter A *Myo-*Inositol and sucrose concentrations affect the accumulation of raffinose family oligosaccharides in seeds J Exp Bot 2004 55 405 1981 1987 10.1093/jxb/erh216 15286144

[B30] Peters S Mundree SG Thomson JA Farrant JM Keller F Protection mechanisms in the resurrection plant *Xerophyta viscosa *(Baker): both sucrose and raffinose family oligosaccharides (RFOs) accumulate in leaves in response to water deficit J Exp Bot 2007 58 8 1947 1956 10.1093/jxb/erm056 17452754

[B31] Alcazar-Roman AR Wente SR Inositol polyphosphates A new frontier for regulating gene expression Chromosoma 2008 117 1 13 10.1007/s00412-007-0126-4 17943301

[B32] Majumder AL Johnson MD Henry SA 1L-myoinositol-1-phosphate synthase Biochim Biophys Acta 1997 1348 1-2 245 256 10.1016/S0005-2760(97)00122-7 9370339

[B33] Parthasarathy L Vadnal RE Parthasarathy R Devi CS Biochemical and molecular properties of lithium-sensitive *myo-*inositol monophosphatase Life Sci 1994 54 16 1127 1142 10.1016/0024-3205(94)00835-3 8152337

[B34] Matsumura H Yoshida K Luo S Kimura E Fujibe T Albertyn Z Barrero RA Krüger DH Kahl G Schroth GP Terauchi R High-throughput superSAGE for digital gene expression analysis of multiple samples using next generation sequencing PLoS ONE 2010 5 8 e12010 10.1371/journal.pone.0012010 20700453 PMC2917361

[B35] GENOSOJA database (Brazilian Soybean Genome Consortium) http://bioinfo03.ibi.unicamp.br/soja 10.1590/S1415-47572012000200001 PMC3392889 22802722

[B36] Benko-Iseppon AM Nepomuceno AL Abdelnoor RV GENOSOJA - The Brazilian soybean genome consortium: high throughput omics and beyond Genet Mol Biol 2012 35 2 i iv 22802722 10.1590/S1415-47572012000200001 PMC3392889

[B37] Phytozome http://www.phytozome.net/

[B38] Kotchoni SO Jimenez-Lopez JC Kayodé AP Gachomo EW Baba-Moussa L The soybean aldehyde dehydrogenase (ALDH) protein superfamily Gene 2012 495 2 128 33 10.1016/j.gene.2011.12.035 22226812

[B39] Ishitani M Nakamura T Han SY Takabe T Expression of the betaine aldehyde dehydrogenase gene in barley in response to osmotic stress and abscisic acid Plant Mol Biol 1995 27 2 307 15 10.1007/BF00020185 7888620

[B40] Nakamura T Nomura M Mori H Jagendorf AT Ueda A Takabe T An isozyme of betaine aldehyde dehydrogenase in barley Plant Cell Physiol 2001 42 10 1088 1092 10.1093/pcp/pce136 11673624

[B41] Chen JB Wang SM Jing RL Mao XG Cloning the PvP5CS gene from common bean (*Phaseolus vulgaris*) and its expression patterns under abiotic stresses J Plant Physiol 2009 166 1 12 9 10.1016/j.jplph.2008.02.010 18565618

[B42] Delauney AJ Verma DP A soybean gene encoding delta 1-pyrroline-5-carboxylate reductase was isolated by functional complementation in *Escherichia coli *and is found to be osmoregulated Mol Gen Genet 1990 221 3 299 305 2199815 10.1007/BF00259392

[B43] Williamson CL Slocum RD Molecular cloning and evidence for osmoregulation of the delta 1-pyrroline-5-carboxylate reductase (proC) gene in pea (*Pisum sativum *L.) Plant Physiol 1992 100 1464 1470 10.1104/pp.100.3.1464 11537868 PMC1075807

[B44] Simon-Sarkadi L Kocsy G Várhegyi A Galiba G de Ronde JA Genetic manipulation of proline accumulation influences the concentrations of other aminoacids in soybean subjected to simultaneous drought and heat stress J Agric Food Chem 2005 53 19 7512 7517 10.1021/jf050540l 16159180

[B45] Kaur H Shukla RK Yadav G Chattopadhyay D Majee M Two divergent genes encoding L-myo-inositol 1-phosphate synthase1 (CaMIPS1) and 2 (CaMIPS2) are differentially expressed in chickpea Plant Cell Environ 2008 31 11 1701 1716 10.1111/j.1365-3040.2008.01877.x 18721262

[B46] Sato Y Yazawa K Yoshida S Tamaoki M Nakajima N Iwai H Ishii T Satoh S Expression and functions of *myo*-inositol monophosphatase family genes in seed development of Arabidopsis J Plant Res 2011 124 3 385 394 10.1007/s10265-010-0381-y 20960216

[B47] Li HW Zang BS Deng XW Wang XP Overexpression of the trehalose-6-phosphate synthase gene OsTPS1 enhances abiotic stress tolerance in rice Planta 2011 234 5 1007 1018 10.1007/s00425-011-1458-0 21698458

[B48] Ge LF Chao DY Shi M Zhu MZ Gao JP Lin HX Overexpression of the trehalose-6-phosphate phosphatase gene OsTPP1 confers stress tolerance in rice and results in the activation of stress responsive genes Planta 2008 228 1 191 201 10.1007/s00425-008-0729-x 18365248

[B49] Oliveira ARS Brasileiro-Vidal AC Bortoleti KCA Bezerra-Neto JP Abdelnoor RV Benko-Iseppon AM Mining plant genome browsers as a mean for efficient connection of physical, genetic and cytogenetic mapping: an example using soybean Genet Mol Biol 2012 35 1 335 347 (Suppl 1) 10.1590/S1415-47572012000200015 22802719 PMC3392886

[B50] Schmutz J Cannon SB Schlueter J Ma J Mitros T Hyten DL Song Q Thelen JJ Cheng J Xu D Hellsten U May GD Yu Y Sakurai T Umezawa T Bhattacharyya MK Sandhu D Valliyodan B Lindquist E Peto M Grant D Shu S Goodstein D Barry K Futrell-Griggs M Abernathy B Du J Tian Z Zhu L Genome sequence of the paleopolyploid soybean Nature 2010 463 178 183 10.1038/nature08670 20075913

[B51] Wanderley-Nogueira AC Soares-Cavalcanti NM Belarmino LC Bezerra-Neto JP Kido EA Pandolfi V Abdelnoor R Bineck E Carazzole MF Benko-Iseppon AM An overall evaluation of the Resistance (R) and Pathogenesis Related (PR) superfamilies in soybean, as compared with Medicago and Arabidopsis Genet Mol Biol 2012 35 1 260 271 (Suppl 1) 10.1590/S1415-47572012000200007 22802711 PMC3392878

[B52] The Arabidopsis Genome Initiative Analysis of the genome sequence of the flowering plant *Arabidopsis thaliana* Nature 2000 408 796 815 10.1038/35048692 11130711

[B53] Shultz JL Kurunam D Shopinski K Iqbal MJ Kazi S Zobrist K Bashir R Yaegashi S Lavu N Afzal JA Yesudas CR Kassem MA Wu C Zhang HB Town CD Meksem K Lightfoot DA The soybean genome database (soyGD): a browser for display of duplicated, polyploidy, regions and sequence tagged sites on the integrated physical and genetic maps of *Glycine max* Nucleic Acids Res 2006 34 1 D758 D756 10.1093/nar/gkj050 16381975 PMC1347413

[B54] Liu Q Wang H Zhang Z Wu J Feng Y Zhu Z Divergence in function and expression of the NOD26-like intrinsic proteins in plants BMC Genomics 2009 10 313 10.1186/1471-2164-10-313 19604350 PMC2726226

[B55] PubMed Database http://www.ncbi.nlm.nih.gov/pubmed

[B56] National Center for Biotechnology Information (NCBI) http://www.ncbi.nlm.nih.gov/

[B57] Matsumura H Kruger DH Kahl G Terauchi R SuperSAGE: a modern platform for genome-wide quantitative transcript profiling Curr Pharm Biotechnol 2008 9 5 368 374 10.2174/138920108785915157 18855689

[B58] Soares-Cavalcanti NM Belarmino LC Kido EA Wanderley-Nogueira AC Bezerra-Neto JP Cavalcanti-Lira R Pandolfi V Nepomuceno AL Abdelnoor RV Nascimento LC Benko-Iseppon AM Overall picture of expressed Heat Shock Factors in *Glycine max, Lotus japonicus *and *Medicago truncatula* Genet Mol Biol 2012 35 2 247 259 22802710 10.1590/S1415-47572012000200006 PMC3392877

[B59] Robertson NM Oveisi-Fordorei SD Varhol RJ Fjell C Marra M Jones S Siddiqui A DiscoverySpace: an interactive data analysis application Genome Biol 2007 8 1 R6 10.1186/gb-2007-8-1-r6 17210078 PMC1839122

[B60] Altschul SF Gish W Miller W Myers EW Lipman DJ Basic local alignment search tool J Mol Biol 1990 215 3 403 410 2231712 10.1016/S0022-2836(05)80360-2

[B61] Krzywinski M Schein J Birol I Connors J Gascoyne R Horsman D Jones SJ Marra MA Circos: an information aesthetic for comparative genomics Genome Res 2009 19 1639 1645 10.1101/gr.092759.109 19541911 PMC2752132

